# Demographic and Clinical Characteristics of Persons With Spinal Cord Injury in Bangladesh: Database for the International Spinal Cord Injury Community Survey 2023

**DOI:** 10.1089/neur.2023.0040

**Published:** 2023-09-06

**Authors:** Taslim Uddin, Mohammad Tariqul Islam, Mohammad Hossain, Mohammad Sohrab Hossain, A.K.M. Salek, M. Jahidul Islam, Shahidul Haque, Hasna Raihan Rahim, Md. Shahadat Hossain, Md. Hassanuzzaman, Monirul Islam, Moin Uddin Hossain Khan, Sharif Ahmed, Khurshid Mahmud, Md. Rakibul Hasan, Anika Tasnim, M. Atiqul Haque

**Affiliations:** ^1^ Department of Physical Medicine and Rehabilitation, Bangabandhu Sheikh Mujib Medical University, Dhaka, Bangladesh.; ^2^ Department of Neurosurgery, Bangabandhu Sheikh Mujib Medical University, Dhaka, Bangladesh.; ^3^ Centre for the Rehabilitation of the Paralysed, Savar, Dhaka, Bangladesh.; ^4^ Department of Physical Medicine and Rehabilitation, Dhaka Medical College, Dhaka, Bangladesh.; ^5^ Department of Physical Medicine and Rehabilitation, Combined Military Hospital, Dhaka, Bangladesh.; ^6^ Department of Physical Medicine and Rehabilitation, Shaheed Suhrawardy Medical College, Dhaka, Bangladesh.; ^7^ Department of Neurology, Chittagong Medical College Hospital, Chattogram, Bangladesh.; ^8^ Department of Physical Medicine and Rehabilitation, National Institute of Traumatology and Orthopedic Rehabilitation, Dhaka, Bangladesh.; ^9^ Department of Physical Medicine and Rehabilitation, National Institute of Neurosciences and Hospital, Dhaka, Bangladesh.; ^10^ Department of Public Health and Informatics, Bangabandhu Sheikh Mujib Medical University, Dhaka, Bangladesh.

**Keywords:** Bangladesh, clinical characteristics, demography, spinal cord injury

## Abstract

The study aims to explore the demographic and clinical characteristics of persons with spinal cord injury (SCI) in Bangladesh. A total of 3035 persons with SCI spanning from 2018 to 2022 were included in this cross-sectional study. Information about demographic and clinical variables was obtained from the medical records and verified through telephone calls to ensure accuracy and consistency. Approximately half (48.30%) of the study participants were located in Dhaka Division. The average age of persons with SCI was 38.3 years, with a standard deviation of 15.9 years, and the largest proportion (33.4%) fell within the age range of 18–30 years. Males outnumbered females by nearly 2.5 times. In the study, 59.6% had suffered traumatic injuries, whereas 40.4% had SCI attributable to disease-related causes; 58.1% were diagnosed with tetraplegia and 40.1% with paraplegia. Fall from height (42.1%) and road traffic trauma (27%) were the most common causes of traumatic injuries. Degenerative myelopathy (41.1%) was the most frequent cause of non-traumatic SCI, followed by tumors (27.7%) and tuberculosis (TB; 14.8%). Both traumatic (58.3%) and degenerative (56.7%) causes of SCI commonly affected the cervical spine, whereas TB (24.4%) and tumors (47.5%) had a higher incidence of affecting the dorsal spine. In the absence of a registry or national database for patients with SCI in Bangladesh, this study would serve as representative data for future studies.

## Introduction

Spinal cord injury (SCI) is a significant global health concern that disproportionately affects persons living in low- and middle-income countries, particularly in Bangladesh.^1–3^ Despite the high incidence of SCI in Bangladesh, there is limited information available on the demographic and clinical characteristics of persons with SCI in this country. Most of the previous studies on SCI in Bangladesh have been single-center based or outdated, and there is no national registry or database for persons with SCI in Bangladesh.^1,4–6^

The lack of standardized essential data for classifying and comparing SCI with other countries remains a significant concern. The classification and comparison of SCI require a standardized essential data set, which includes basic demographic characteristics, initial acute and rehabilitation care, cause of injury, presence of vertebral fractures, and associated injuries.^7^ However, such a standardized data set does not exist in Bangladesh, and this has limited the ability to understand the epidemiology of SCI and provide appropriate treatment and rehabilitation services.^7^

According to an empirical study, the total number of persons with SCI was found to be 1035 in 2019 in Bangladesh. The study revealed that the majority of subjects were hospitalized by non-government facilities (38.65%), whereas only two government hospitals accounted for roughly 30.0% of SCI subjects.^4^ However, this study had a limited scope given that it covered only two hospitals. Therefore, to address this research gap, the current study aims to investigate the demographic and clinical characteristics to cover all the major public and private medical facilities that cater to persons with SCI in Bangladesh.

By gathering comprehensive data on persons with SCI, including contact address and phone number, geographical locations, etiological patterns, trauma types, or communal experiences, this study aims to establish a representative registry or database for persons with SCI in Bangladesh.^8^ This registry or database would facilitate the planning and provision of appropriate medical and rehabilitation services, as well as promote research on SCI in Bangladesh.

## Methods

A study center (BanSCI) comprised of physiatrists, neurologists, spinal neurosurgeons, physiotherapists, and public health specialists was established in Bangladesh in 2020. In a consensus meeting, the study center identified 18 tertiary referral hospitals, rehabilitation facilities, and specialty care clinics that were known to serve most of the persons with SCI in Bangladesh.

In this cross-sectional study, data from a total of 5000 documented patients diagnosed with SCI were retrieved from the medical records of the selected 18 hospitals between 2018 and 2022. The study included persons with both traumatic and non-traumatic SCI, as well as those with cauda equina syndrome while excluding those who could not communicate or had SCI attributable to congenital causes such as spina bifida, motor neuron disease, multiple sclerosis, or peripheral nerve damage like Guillain-Barre syndrome or poliomyelitis. Basic demographic and clinical information, including address and cell phone number, was collected from the medical records using a semistructured questionnaire.

After the initial screening, 3035 persons with SCI were included in the study. Ethical approval was obtained from the Institutional Review Board of Bangabandhu Sheikh Mujib Medical University, Dhaka, Bangladesh (reference no.: 2021-12826), as well as approval to use hospital records from the respective hospital directors and the Directorate General of Health Services of Bangladesh. Four medical graduates were recruited and underwent a 7-day training session focused on several key aspects, including data collection from hospital records, effective communication with persons with SCIs, obtaining verbal consent, and ensuring the utmost patient privacy while cross-checking data over the phone.

### Statistical analysis

Participants' characteristics (age, sex, causes of injury, and vertebral injuries) and injury characteristics (location of injury and completeness of injury) were analyzed descriptively. Continuous variables are expressed as mean with standard deviation (SD) and median with range. Categorical variables are expressed as the number of cases and percentages. For age, two sets of categories were created. The Statistical Package for Social Sciences (SPSS-26; SPSS, Inc., Chicago, IL) was used for data analysis.

## Results

### Geographical distribution across the administrative divisions of the country

**FIG. 1.**
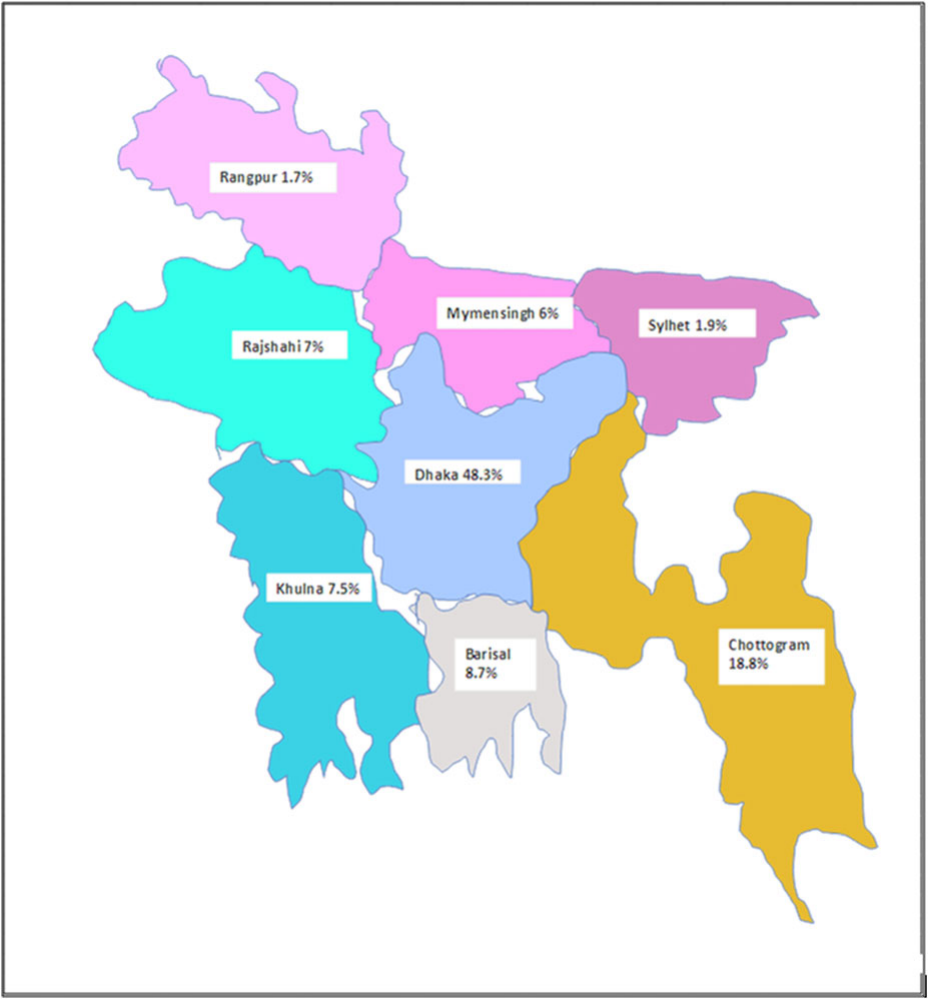
Geographical distribution at different divisions of persons with SCI in Bangladesh. In Dhaka 48.3%, Chattogram 18.8%, Barisal 8.7%, Khulna 7.5%, Rajshahi 7%, Rangpur 1.7%, Mymensingh 6%, and Sylhet 1.9% persons are suffering from SCI. SCI, spinal cord injury. (This figure is for illustrative purposes only and does not represent the actual map of Bangladesh.)

Figure 1 illustrates the distribution of study participants according to their respective divisions. Dhaka Division accounted for ∼48.30% of participants, making it the most prominently represented division. Chattogram followed with 18.8%, Barisal with 8.7%, Khulna with 7.5%, and Rajshahi with 7% of participants. Notably, the coastal southern divisions of Chattogram (18.8%), Barisal (8.7%), and Khulna (7.5%) exhibited a higher prevalence of persons with SCI compared to the northern divisions, presenting an intriguing finding.

### Age and sex distribution of the persons with spinal cord injury

**FIG. 2.**
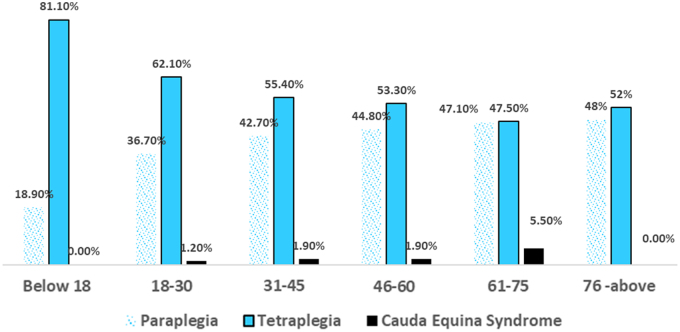
Distribution of subjects according to age group and clinical diagnosis (*n* = 3035). The bar chart displays the three clinical diagnostic features of persons with SCI according to their age group. These three are: paraplegia, tetraplegia, and cauda equina syndrome. Each bar represents a definite age group (<18 years, 18–30 years, 31–45 years, 46–60 years, 61–75 years, and the ≥76 age group). SCI, spinal cord injury.

The study population was divided into six groups: <18 years; 18–30 years; 31–45 years; 46–60 years; 61–75 years; and the ≥76 age group, respectively. Persons with SCI were predominantly 18–30 years of age (33.4%), followed by 31–45 (29.2%) and 46–60 years (22.6%), with a mean age of 38.3 years (SD = 15.9), as shown in Figure 2. There were fewer persons with SCI <18 (6.1%) and ≥76 years of age (0.8%). There were 71.4% males and 28.6% females, with a male-female ratio of 2.5:1.

### Cause of spinal cord injury

Among the 3035 persons with SCI, 1808 (59.6%) had traumatic injuries, whereas 1227 (40.4%) had SCI attributable to disease-related causes.

### Traumatic cause of spinal cord injury

The most common causes of traumatic SCI were fall from height (42.1%) and road traffic accidents (RTAs; 27%), followed by an attempted suicide and other less common causes such as carrying heavy objects, falling heavy objects, and accidental strangulation because of a scarf injury (Table 1). Other causes of traumatic SCI included iatrogenic surgical trauma, work-related trauma, household trauma, bull attack, and other undetermined causes.

**Table 1.** Frequency of Traumatic and Disease-Related Cause of SCI (*n* = 3035)

Causes of SCI	Frequency	Percentage
Traumatic (*n* = 1808)		
Fall from height	761	42.1
Road traffic accidents	489	27.0
Attempted suicide	188	10.4
Carrying heavy object	73	4.0
Fall of heavy object	45	2.5
Accidental strangulation	25	1.4
Violence	14	0.8
Sports	7	0.4
Others (surgical, work related, household, bull attack)	206	11.4
Disease related (*n* = 1227)		
Degenerative myelopathy	504	41.1
Tumor (benign and malignant)	340	27.7
TB	181	14.8
Infection (viral, bacterial)	46	3.7
TM (autoimmune)	22	1.8
Vascular (AVM)	9	0.7
Miscellaneous	125	10.2

AVM, arteriovenous malformation; SCI, spinal cord injury; TB, tuberculosis; TM, transverse myelitis.

### Non-traumatic cause of spinal cord injury

The leading cause of disease-related SCIs is degenerative myelopathy (DM), accounting for 41.1% of all cases, as indicated in Table 1. Tumors were the second most common cause, accounting for 27.7% of cases. In this study, 14.8% of participants had SCI caused by tuberculosis (TB; Pott's spine). Though less common, non-specific infection, transverse myelitis (TM), and vascular abnormalities were also observed as causes of SCI. Additionally, various causes accounted for 10.2% of cases, where the medical records or follow-up phone calls could not determine the disease or disorder that led to SCI.

### Spinal level of spinal cord injury and relation with the causes

Spinal level of SCI with its relation to etiology is presented in Table 2.

**Table 2.** Spinal Level of Injury According to SCI Etiology (*n* = 3035)

	Cervical spine (*n* = 1770)	Dorsal spine (*n* = 690)	Lumbar spine (*n* = 575)
Traumatic (*n* = 1808)			
RTA	265 (23.7)	130 (37.1)	94 (27.6)
Fall from height	343 (30.7)	207 (59.1)	211 (62.1)
Sports	6 (0.5)	1 (0.3)	0 (0)
Violence	6 (0.5)	5 (1.4)	3 (0.9)
Attempted suicide	186 (16.6)	0 (0)	2 (0.6)
Carrying heavy object	68 (6.1)	3 (0.9)	2 (0.6)
Accidental strangulation because of scarf injury	25 (2.2)	0 (0)	0 (0)
Others (surgical, work related, household, bull attack)	178 (15.9)	0 (0)	28 (8.2)
Fall of heavy object	41 (3.7)	4 (1.1)	0 (0)
Disease related (*n* = 1227)			
TB	57 (8.7)	83 (24.4)	41 (17.4)
Tumor	113 (17.3)	162 (47.6)	65 (27.7)
Infection	16 (2.5)	6 (1.8)	24 (10.2)
DM	370 (56.7)	61 (17.9)	73 (31.1)
TM (autoimmune)	6 (0.9)	8 (2.4)	8 (3.4)
Vascular (AVM)	5 (0.8)	4 (1.2)	0 (0)
Miscellaneous	85 (13)	16 (4.7)	24 (10.2)

AVM, arteriovenous malformation; DM, degenerative myelopathy; RTA, road traffic accident; SCI, spinal cord injury; TB, tuberculosis; TM, transverse myelitis.

The most prevalent level of injury in persons with traumatic SCIs was cervical, accounting for 58.3% of cases, followed by thoracic at 22.7% and lumbar at 18.8%. Road traffic trauma and falls from height were the leading traumatic causes of dorsal spine SCI, followed by the cervical and lumbar spine. In cases of suicide attempts by hanging, the cervical spine was mainly involved among the 186 persons with SCI.

DM was the most common disease-related cause of SCI, with a higher prevalence of cervical spine involvement (56.7%) compared to the lumbar (31.1%) and thoracic spine (17.9%). TB (24.4%) and tumor (47.6%) involved the dorsal spine more frequently compared to the cervical and lumbar spines.

Figure 2 shows that 40.1% of persons with SCI were diagnosed with paraplegia, whereas 58.1% were diagnosed with tetraplegia, and the remaining 1.8% were diagnosed with cauda equina syndrome. Information regarding severity of SCI lesion was available for 29.4% of participants. Of these, 63% had incomplete lesions, whereas 37% had complete lesions (according to the American Spinal Injury Association Impairment Scale [AIS] classification system). Among those with incomplete lesions, AIS-D was the most common (44.2%), followed by AIS-C (10.8%).

One hundred thirty-nine patients had additional injuries, such as limb, head, or rib fractures, whereas 48 persons with SCI had pressure injuries. Bowel and bladder involvement were found in 201 persons with SCI. It could be attributable to the unavailability of data. We tried our best to get the information, but this much was available during data analysis.

## Discussion

In this study, it was observed that the majority of persons diagnosed with SCI (48.3%) were residing in Dhaka Division. This can be attributed to the notable concentration of tertiary care government or university-level hospitals in Dhaka, which frequently handle complex medical cases. Additionally, the maldistribution of persons with SCI in this study can be explained by the unequal distribution of healthcare professionals between urban and rural areas in Bangladesh. It is worth noting that 61% of the population resides in rural regions, whereas a greater number of healthcare professionals are concentrated in urban areas, such as the capital city of Dhaka.^9^

In the current study, the male-female ratio was 5:2. It is important to note that this ratio is specific to the sample of patients selected from the hospital registry and may not reflect the overall distribution of persons with SCI. Nevertheless, numerous studies have consistently indicated a male predominance in SCI cases.^1,8,10^ Specifically, when examining global data, Ethiopia exhibited the highest male-to-female ratio at 7.6:1, whereas Turkey had the lowest ratio at 1.6:1.^11^

In this study, ∼60% of patients had a traumatic history, whereas 40% had non-traumatic, disease-related SCI. However, a previous hospital-based study conducted in Bangladesh reported a significantly lower proportion of non-traumatic cases, at only 15%.^5^ It is worth noting that non-traumatic causes of SCI are becoming more prevalent as the population ages.^12^

Among traumatic cases, falling from heights was found to be the most common cause of SCI in males, followed by RTAs, accounting for 27.5% of cases. There was a notable difference between the sexes, with 29.0% of males and 23.0% of females being affected by RTAs. Another Bangladeshi study reported that falls accounted for 50.5% of cases, whereas RTAs accounted for 26.0%.^4^ Additionally, an emerging cause of SCI in Bangladesh is attempted suicide by strangulation and scarf strangulation, which is predominantly observed in the younger age group.^13^

In this study, 58% of participants had tetraplegia, and among them, 63% had incomplete spinal cord lesions. This indicates a higher frequency of cervical spinal involvement, particularly degenerative compressive myelopathy, compared to earlier studies.^4,14^ In addition to this, other causes of tetraplegia were traumatic injuries, tumors affecting the spinal cord, or TB-related complications. These factors contribute to the variation in the prevalence of tetraplegia and paraplegia cases observed in the current study.

The strength of the study lies in its novelty and comprehensiveness. Being the first study of its kind in Bangladesh, it provides important insights into the characteristics and needs of persons with SCI in the country. The study included multiple centers and used updated contacts for follow-up, which is also a strength, given that it allows for a more representative sample and more reliable data. This study also provides valuable information that can be used to improve care and support for persons with SCI in Bangladesh and can serve as a basis for further research and intervention in this area.

The study possesses several limitations that warrant acknowledgment. First, the study heavily relied on manual searches of paper-based hospital records, resulting in the absence of certain information or its unavailability within the records. Notably, details regarding pressure injuries, bowel-bladder status, AIS classification, and demographic characteristics were inadequately documented for some participants. Further, crucial information, such as the spinal level of injury or the cause of injury, was missing from the medical records and had to be obtained through telephone interviews, but it could not be cross-verified for accuracy. These limitations may have resulted in an incomplete or potentially inaccurate portrayal of the demographic and clinical profile of persons with SCI in Bangladesh. Future studies should strive to incorporate more comprehensive data collection methods in order to address these limitations effectively.

## Conclusion

This study presents a comprehensive data set on the demographic and clinical characteristics of persons with SCI in Bangladesh. The study involved multiple centers, and the data set was obtained through a combination of an updated addressograph, medical records, and telephone interviews to ensure accuracy and consistency. The study provides valuable insights into the epidemiology of SCI in Bangladesh, including the age distribution, sex ratio, and etiology of SCI, which are essential for the development of appropriate prevention and management strategies. The data set generated from this study will serve as a vital resource for future research on SCI in Bangladesh and provide a basic registry of persons with SCI for strategic welfare planning. This study highlights the need for a national registry or database for SCI in Bangladesh to better understand and address the needs of this population.

## Abbreviations Used

AIS: American Spinal Injury Association Impairment Scale
DM: degenerative myelopathy
RTAs: road traffic accidents
SCI: spinal cord injury
SD: standard deviation
TB: tuberculosis
TM: transverse myelitis
